# Strengthening Performance Optimization of Single Ball Impact Treatment by Evaluating Residual Stress

**DOI:** 10.3390/ma15103719

**Published:** 2022-05-23

**Authors:** Yang Lei, Zhengwei Wang, Huan Qi

**Affiliations:** 1 Special Equipment Institute, Hangzhou Vocational & Technical College, Hangzhou 310018, China; 2017010004@hzvtc.edu.cn; 2 College of Mechanical Engineering, Zhejiang University of Technology, Hangzhou 310023, China; huanqi@zjut.edu.cn

**Keywords:** strengthening performance, residual stress, single ball impacts, high manganese steel

## Abstract

Residual stress plays an essential role in affecting the strengthening performance by a surface treatment. Studying the impact of a single ball on a target surface is the basis of shot peening technology in order to obtain the distribution of the residual stress, and to optimize the processing parameters. In this paper, a numerical model has been developed to represent single ball impacts on high manganese steel by considering different processing parameters. It was found that by increasing the ball diameter and impact velocity, the depth of maximum residual compressive stress and the depth of the residual compressive stress layer became significantly enlarged due to increasing kinetic energy of the impacting ball. It was also found from simulation that with an increase in ball impact angle, the maximum residual compressive stress, the depth of maximum residual compressive stress and the depth of the residual compressive stress layer were significantly improved; an exception was the surface residual compressive stress, which showed a decreasing trend. Moreover, by employing quantitative analysis with the entropy method, it was found that within the range of processing parameters considered in the simulation, it is recommended to use a ball with a diameter of 0.6 mm to impact the target, with a velocity of 80 m/s and an angle of 90° for the best strengthening performance.

## 1. Introduction

Surface treatment is a process of artificially forming a hardened layer on the target surface by using induced residual stress in order to forge a material that has different properties from the mechanical, physical and chemical properties of the original target material [1,2,3]. The purpose of surface treatment is to meet the requirements of corrosion resistance, wear resistance or other special functions of the product, not only to enhance the bearing capacity of the equipment, but also to prolong its service life [4]. Currently, many surface treatment methods, such as laser shock processing, mechanical rolling and shot peening, have been extensively employed to strengthen mechanical parts with different structures [5,6,7]. With the assistance of ultrasonic vibration, ultrasonic shot peening and ultrasonic nanocrystal surface modification technologies have also been proposed to enhance the strengthening performance, due to the formation of nanocrystal structures on the target surface [8,9]. Shot peening technology is mainly carried out using a shot peening machine in order to drive a ball impacting on the target surface at a certain velocity and angle, which can induce residual stress from the target’s surface to subsurface with a certain depth; in this way, it can prolong the fatigue life or load capacity of the target, and improve its fatigue strength and corrosion resistance performance as well [10,11,12]. With the development of science and technology, many researchers investigate the shot peening process and its strengthening mechanism in order to improve the accuracy and efficiency of the shot peening equipment; thus, shot peening technology has been developing rapidly. Bozkurt et al. [13] employed the shot peening of AISI 4140 low-alloy steel and found that the tribocorrosion performance of the material improved with increasing shot peening intensity, in terms of increased surface hardness. The distribution of residual stress in shot peening of 12Cr2Ni4A steel was investigated by Zhao et al. [11] in order to optimize the processing parameters, and a numerical stimulation using the Computational Fluid Dynamics and Finite Element Model was also conducted in order to study the influence of processing parameters on the induced compressive residual stress and its strengthening mechanism [14]. Residual stress is one of the most important factors affecting machining performance in the advanced manufacturing process [15,16], and should be considered in the shot peening process as well.

The selection and optimization of processing parameters in the shot peening process have an important influence on the distribution of residual stress on target materials, and hence their strengthening performance. In general, the shot peening process is associated with many processing parameters, and its strengthening mechanism is also complicated. Thus, it is difficult to realize the relationship between processing parameters and the shot peening performance directly with the mathematical model, which usually depends on many tests that are not only time-consuming but also expensive. Numerical simulation is a powerful tool used to study a wide range of complicated problems that cannot be addressed experimentally; it normally does not require as many assumptions as the mathematical method, and can reveal more about detailed processes that cannot be obtained experimentally [17,18,19,20].

The model of single ball impacts is the basis of the numerical simulation of the shot peening process, and has small calculation requirements which can be used to analyze the influence of various processing parameters on the strengthening performance. Meguid et al. [21] established a single ball impacting model to study the influence of ball velocity, size and shape on the residual stress of target materials, and the depth of the residual stress layer. Kim et al. [22] studied the formation of residual stress on targets after the shot peening process by modelling the impacts of single ball. Hong et al. [23] used the Finite Element Method to establish the numerical model of a single ball impacting on a target surface; the influence of ball impact velocity and angle on the distribution of the residual compressive stress was analyzed. Wang [24] used LS-DYNA (ANSYS, USA) to establish a single ball impacting model, and designed an orthogonal experiment to analyze the residual compressive stress field of the target under different shot peening processing parameters; an optimal combination of shot peening processing parameters was found. However, limited investigations have been conducted to explore a method to quantitatively evaluate the residual stress induced by the shot peening process, to optimize the processing parameters, and hence its effect on the strengthening performance.

Thus, in this paper a numerical model will first be developed to represent the single ball impacting on high manganese steel. Then, by considering different combinations of ball diameter, impact velocity and impact angle, the numerical tests will be conducted. Four essential factors and values related to the residual stress after the single ball impacts will be numerically obtained in order to analyze the effect of the processing parameters, qualitatively and quantitatively, on the residual stress. The optimized combination of processing parameters will be finally recommended for the best strengthening performance.

## 2. Model Development

**Figure 1:**
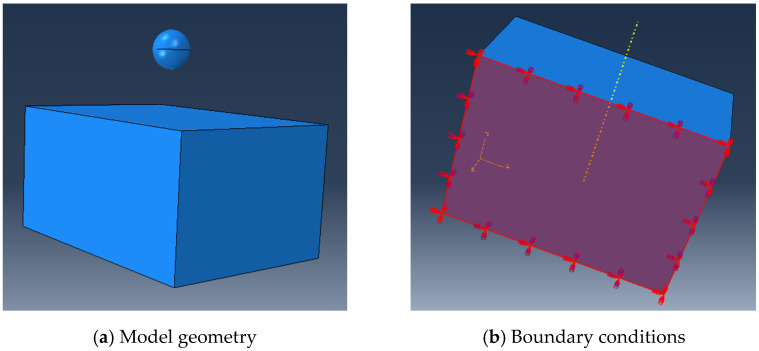
Model geometry and boundary conditions.

For the modelling of the single ball impacts, as shown in Figure 1, the model geometry and boundary conditions are set as follows: the target with dimensions of 4 mm × 3 mm × 2 mm is modelled in ABAQUS (SIMULIA, Dassault Systèmes, Johnston, RI, USA), and the single ball is generated up to the target surface with a distance of 1 mm, as shown in Figure 1a, where the bottom of the target is set as constraints (see Figure 1b). In simulation, high manganese steel with a density of 7.98 g/cm^3^, elastic modulus of 210 GPa and Poisson ratio of 0.3 is selected as the target material. The material of the impacting ball is steel, with a density of 7.8 g/cm^3^, elastic modulus of 210 GPa and Poisson ratio of 0.3. Since shot peening technology is a surface-strengthening method that can effectively enhance the ability of mechanical parts to resist fatigue and wear failure by inducing residual stress and large plastic deformation with a high strain rate, the Johnson–Cook (J-C) model is usually used to numerically model this process, since it is an empirical constitutive model that can describe the strain rate strengthening effect and temperature softening effect of metals [25]. The J-C model can be expressed as follows:

(1) $$\sigma =\left(A+B\varepsilon_{p}^{n}\right)\left(1+C\ln{\dot{\varepsilon }}^{*}\right)\left(1-T^{*m}\right)$$

where *A* is the yield strength by considering the strain rate and temperature; *B* is the hardening modulus of material; *n* is the material hardening coefficient; *C* is the material strain rate strengthening parameter; and m is the temperature susceptibility factor. According to [26], the J-C model parameters for high manganese steel are provided in Table 1, and the composition of high manganese steel (% by mass) used in simulation is shown in Table 2.

In order to improve the simulation efficiency and accuracy, the mesh independence test needs to be conducted. The target is divided into mesh sizes of 0.02 mm, 0.015 mm and 0.01 mm, and it is found that the error of numerical results between adjacent mesh densities is less than 2.1%, thus the mesh size of 0.02 mm is selected for this model in order to achieve an accurate solution and reduce the computation time.

## 3. Numerical Work

The ball diameter, ball impact velocity and impact angle are three essential parameters that can affect the strengthening performance [14], and by considering the ability of the commercial shot peening machine in addition to previous studies in the shot peening of high manganese steel [27], in simulation, single balls with diameters (*d_p_*) of 0.2 mm, 0.4 mm and 0.6 mm are employed; three levels of ball impact velocity (*v_p_*), i.e., 40 m/s, 60 m/s and 80 m/s, are selected. Moreover, the ball impact angle could affect the formation of the plastic zone on the target surface; as such, three levels of ball impact angle (*α_p_*), i.e., 30°, 60° and 90°, are also considered in the simulation. Thus, by using a full factorial design of ball diameter, ball impact velocity and impact angle, a total 27 tests will be carried out.

**Figure 2:**
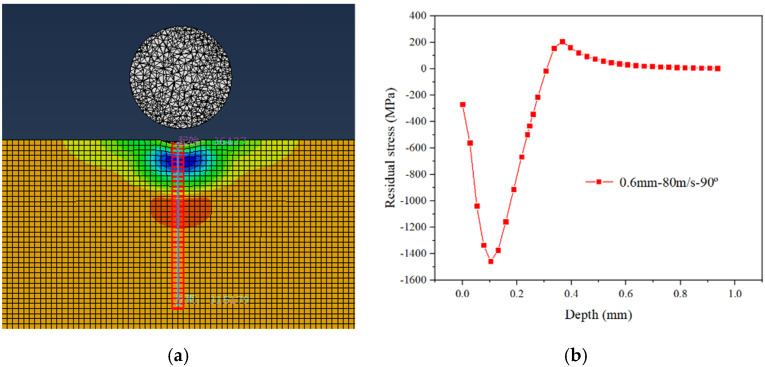
Distribution of residual stress from surface to subsurface of the target. (**a**) Residual stress field; (**b**) residual stress curve.

This study aims to obtain the optimized processing parameters and strengthening performance in the single ball impacting of high manganese steel, by evaluating the induced residual stress in this process. After the impacts of single balls on the target, the residual stress from the target’s surface to subsurface can be generally classified as the surface residual compressive stress (*σ_s_*), the maximum residual compressive stress (*σ_m_*), the depth of maximum residual compressive stress (*Z_m_*) and the depth of residual compressive stress layer (*Z*_0_). These values can be numerically obtained, as can be seen from Figure 2. Figure 2a is a typical residual stress cloud map after the single ball impacting process, using a ball diameter of 0.6 mm, an impact velocity of 80 m/s and an impact angle of 90°; the related residual stress curve can be then obtained, as shown in Figure 2b, where these four essential values mentioned above can be calculated. Thus, quantitative and qualitative analysis will be conducted to optimize the strengthening performance with respect to the different processing parameters by analyzing these four essential values.

## 4. Results and Discussion

### 4.1. Qualitative Analysis

The effects of the processing parameters involved in the single ball impacting process, i.e., ball diameter, ball impact velocity and impact angle, on the distribution of residual stress and the four essential values, are first qualitatively analyzed, as detailed below.

**Figure 3:**
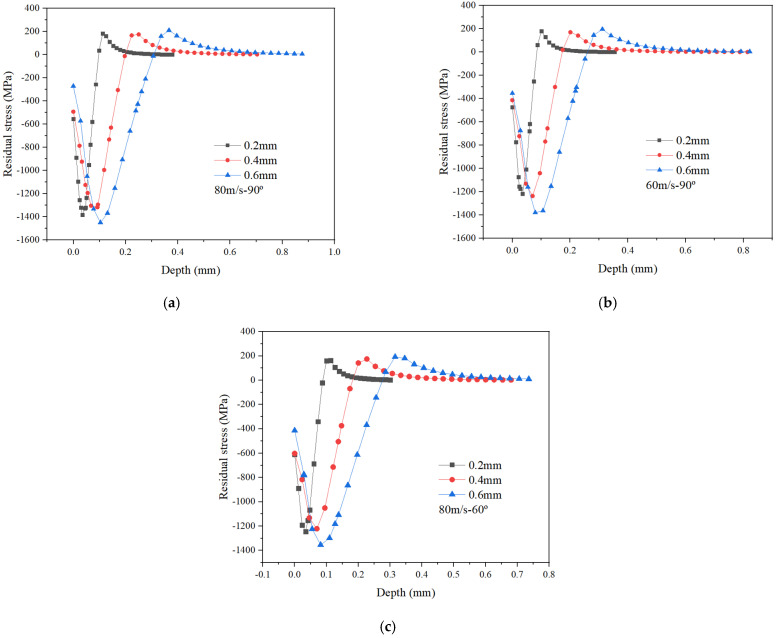
Effect of ball diameter on residual stress under different processing parameters. (**a**) *v_p_* = 80 m/s and *α_p_* = 90°; (**b**) *v_p_* = 60 m/s and *α_p_* = 90°; (**c**) *v_p_* = 80 m/s and *α_p_* = 60°.

It can be seen from Figure 3 that with an increase in ball diameter, the depth of maximum residual compressive stress and the depth of residual compressive stress layer become significantly enlarged, especially the depth of residual compressive stress layer, which extends by more than three times. It can also be seen that the variation in the depth of the residual compressive stress layer is particularly obvious as compared with other values from the residual stress curve. This is because the kinetic energy of the ball increases with the increase in ball diameter, and during the single ball impacting process it can penetrate deeper into the target, resulting a larger residual compressive stress field on the target’s subsurface. Therefore, in order to make the target achieve a deeper residual stress layer, using a ball with a larger diameter is recommended.

**Figure 4:**
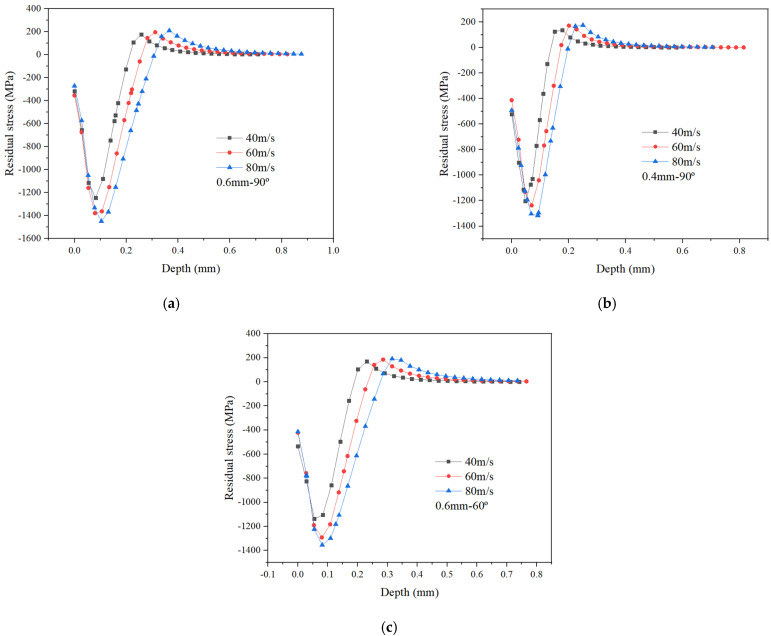
Effect of ball impact velocity on residual stress under different processing parameters. (**a**) *d_p_* = 0.6 mm and *α_p_* = 90°; (**b**) *d_p_* = 0.4 mm and *α_p_* = 90°; (**c**) *d_p_* = 0.6 mm and *α_p_* = 60°.

With an increase in ball impact velocity, the maximum residual compressive stress, the depth of maximum residual compressive stress and the depth of residual compressive stress layer all become enlarged, while the surface residual compressive stress seems to have no obvious change. It is also interesting to note from Figure 4 that the front part of residual stress curve for each group shows little change, with all values close to each other. The rest of the curve shows no obvious change, indicating that the impact velocity has little influence on the residual stress in the shallow surface of the target; however, it has great influence on the target subsurface. Similarly, increasing the impact velocity would also increase the kinetic energy of the ball, which can significantly improve the depth of maximum residual compressive stress and the depth of residual compressive layer over a larger range.

**Figure 5:**
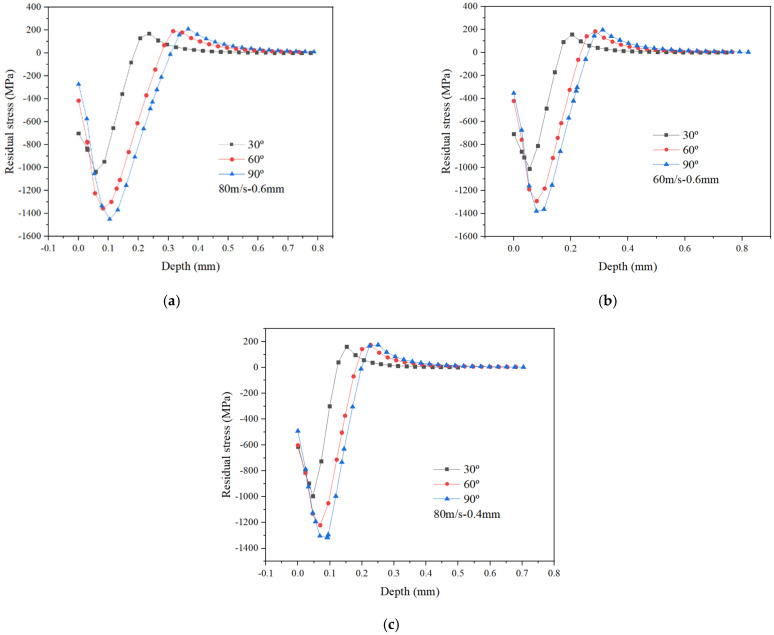
Effect of ball impact angle on residual stress under different processing parameters. (**a**) *v_p_* = 80 m/s and *d_p_* = 0.6 mm; (**b**) *v_p_* = 60 m/s and *d_p_* = 0.6 mm; (**c**) *v_p_* = 80 m/s and *d_p_* = 0.4 mm.

It can be seen from Figure 5 that with an increase in ball impact angle, the maximum residual compressive stress, the depth of maximum residual compressive stress and the depth of residual compressive stress layer are all significantly improved, except for the surface residual compressive stress which shows a decreasing trend. It demonstrates that an increase in ball impact angle produces a significant improvement in the strengthening performance. Therefore, in order to make the target material achieve a better shot peening effect, the ball impact angle should be perpendicular to the target surface.

### 4.2. Quantitative Analysis

In ABAQUS, the distribution of residual stress from the target’s surface to subsurface along its thickness can be observed from each test, and hence, the values of *σ_s_*, *σ_m_*, *Z_m_* and *Z*_0_ can be obtained. as discussed in Section 3. Table 3 shows the relations of these values with respect to different processing parameters; however, note that the units of these four values are different, thus that they cannot be used for direct comparison. Thus, dimensionlessness is employed to process theses values according to the equation as follows:

(2) $$p_{ij}=\frac{x_{ij}}{{\bar{x}}_{j}}\left(i=1,\;2,\;\ldots ,\;27;j=1,\;2,\;3,\;4\right)$$

(3) $${\bar{x}}_{j}=\frac{1}{n}\sum_{i=1}^{n}x_{ij}$$

where, *x_ij_* represents each value and thus, the dimensionless values are shown in Table 4.

The entropy method is one of the weighting methods that has been extensively used to measure the value dispersion in decision-making. It is a measure of linear dependence between a random variable and a set of random variables. If the entropy value of the index is smaller, the amount of information provided by the index is smaller; hence, the role it plays in the comprehensive evaluation is smaller, since its weight is lower. Therefore, the entropy method can be used to calculate the weight of each index and provide a basis for multi-index comprehensive evaluation [28]. Thus, the entropy method is employed in this study to process these dimensionless values in order to evaluate the weighted effect that the residual stress has with respect to different processing parameters based on the following equation that is taken from:

(4) $$S=\overset{4}{\underset{j=1}{\sum }}w_{j}p_{ij}$$

where *w_j_* (*j* = 1, 2, 3, 4) is the weight coefficient which is set to 25% in this study. Hence, the weighted scores from the entropy method are shown in Table 5, and it can be seen from this table that the highest score is 1.4988 in test 27, which indicates that within the range of processing parameters considered in the simulation, it is recommended to use a ball with a diameter of 0.6 mm to impact the target with a velocity of 80 m/s and an angle of 90° for the best strengthening performance.

## 5. Conclusions

In this study, a numerical model has been developed in the ABAQUS with J-C model to represent the single ball impacting on high manganese steel by considering different processing parameters, i.e., ball diameter, ball impact velocity and impact angle, with a full factorial design. The main contributions of this paper can be summarized as follows:

- The values of the surface residual compressive stress, the maximum residual compressive stress, the depth of maximum residual compressive stress and the depth of residual compressive stress layer from the target’s surface to subsurface are four essential factors that affect the surface strengthening performance, and can be numerically obtained rather than by using experimental methods, which are expensive and time-consuming.
- It was found from simulation that by increasing the ball diameter and impact velocity, the depth of maximum residual compressive stress and the depth of residual compressive stress layer become significantly enlarged due to increasing kinetic energy of the shot peening ball. It was also numerically found that with an increase in ball impact angle, the maximum residual compressive stress, the depth of maximum residual compressive stress and the depth of residual compressive stress layer become significantly improved, except for the surface residual compressive stress which shows a decreasing trend.
- Quantitative analysis according to the entropy method was employed to consider the effect of processing parameters on the four essential values of residual stress obtained from simulation. It was found that within the range of processing parameters considered in this simulation, a ball with a diameter of 0.6 mm should be used to impact the target with a velocity of 80 m/s and an angle of 90°; this would subsequently obtain the best strengthening performance in terms of evaluating the distribution of residual stress.

## Figures and Tables

**Table 1:** J-C model parameters of high manganese steel [26].

*A*/MPa	*B*/MPa	*n*	*C*	*m*
634	897	0.913	0.04	1

**Table 2:** The composition of high manganese steel (% by mass).

Mn	C	S_i_	P	S
11–14	1–1.4	0.3–1	<0.03	<0.05

**Table 3:** Original simulation results of residual stress.

No.	*d_p_* (mm)	*v_p_* (m/s)	*α_p_* (°)	Parameters			
				*σ_s_* (MPa)	*σ_m_* (MPa)	*Z_m_* (mm)	*Z*_0_ (mm)
1	0.2	40	30	−674.245	−780.380	0.013	0.044
2	0.2	40	60	−670.691	−1092.080	0.024	0.060
3	0.2	40	90	−586.816	−1194.410	0.025	0.065
4	0.2	60	30	−638.922	−817.008	0.025	0.052
5	0.2	60	60	−520.579	−1117.960	0.024	0.074
6	0.2	60	90	−472.759	−1217.970	0.035	0.081
7	0.2	80	30	−611.712	−1002.980	0.024	0.061
8	0.2	80	60	−609.965	−1244.020	0.035	0.087
9	0.2	80	90	−555.414	−1390.040	0.036	0.093
10	0.4	40	30	−667.563	−757.921	0.025	0.081
11	0.4	40	60	−559.120	−1043.590	0.048	0.125
12	0.4	40	90	−521.545	−1203.650	0.049	0.140
13	0.4	60	30	−645.162	−845.771	0.048	0.110
14	0.4	60	60	−640.605	−1125.830	0.049	0.155
15	0.4	60	90	−413.234	−1237.520	0.070	0.170
16	0.4	80	30	−613.877	−996.326	0.047	0.120
17	0.4	80	60	−601.694	−1221.590	0.070	0.180
18	0.4	80	90	−492.369	−1317.470	0.093	0.201
19	0.6	40	30	−635.010	−891.511	0.058	0.130
20	0.6	40	60	−534.369	−1137.270	0.055	0.185
21	0.6	40	90	−318.198	−1245.100	0.081	0.210
22	0.6	60	30	−706.755	−1011.280	0.055	0.160
23	0.6	60	60	−421.261	−1291.860	0.079	0.231
24	0.6	60	90	−353.639	−1379.460	0.080	0.263
25	0.6	80	30	−700.955	−1036.950	0.057	0.182
26	0.6	80	60	−414.197	−1354.220	0.082	0.271
27	0.6	80	90	−270.975	−1449.210	0.105	0.310

**Table 4:** Dimensionless values of residual stress.

No.	*d_p_* (mm)	*v_p_* (m/s)	*α_p_* (°)	Parameters			
				*σ* *_s_*	*σ_m_*	*Z_m_*	*Z*_0_
1	0.2	40	30	0.3093	0.6930	0.2522	0.3093
2	0.2	40	60	0.4218	0.9698	0.4655	0.4218
3	0.2	40	90	0.4569	1.0607	0.4849	0.4569
4	0.2	60	30	0.3655	0.7256	0.4849	0.3655
5	0.2	60	60	0.5202	0.9928	0.4655	0.5202
6	0.2	60	90	0.5694	1.0816	0.6789	0.5694
7	0.2	80	30	0.4288	0.8907	0.4655	0.4288
8	0.2	80	60	0.6116	1.1048	0.6789	0.6116
9	0.2	80	90	0.6537	1.2344	0.6983	0.6537
10	0.4	40	30	0.5694	0.6731	0.4849	0.5694
11	0.4	40	60	0.8787	0.9268	0.9310	0.8787
12	0.4	40	90	0.9841	1.0689	0.9504	0.9841
13	0.4	60	30	0.7732	0.7511	0.9310	0.7732
14	0.4	60	60	1.0896	0.9998	0.9504	1.0896
15	0.4	60	90	1.1950	1.0990	1.3578	1.1950
16	0.4	80	30	0.8435	0.8848	0.9116	0.8435
17	0.4	80	60	1.2653	1.0848	1.3578	1.2653
18	0.4	80	90	1.4129	1.1700	1.8039	1.4129
19	0.6	40	30	0.9138	0.7917	1.1250	0.9138
20	0.6	40	60	1.3004	1.0100	1.0668	1.3004
21	0.6	40	90	1.4762	1.1057	1.5711	1.4762
22	0.6	60	30	1.1247	0.8981	1.0668	1.1247
23	0.6	60	60	1.6238	1.1472	1.5323	1.6238
24	0.6	60	90	1.8487	1.2250	1.5517	1.8487
25	0.6	80	30	1.2794	0.9209	1.1056	1.2794
26	0.6	80	60	1.9050	1.2026	1.5905	1.9050
27	0.6	80	90	2.1791	1.2870	2.0366	2.1791

**Table 5:** Scores from the entropy method.

No.	*d_p_* (mm)	*v_p_* (m/s)	*α_p_* (°)	Score
1	0.2	40	30	0.6201
2	0.2	40	60	0.7691
3	0.2	40	90	0.7673
4	0.2	60	30	0.6844
5	0.2	60	60	0.7312
6	0.2	60	90	0.7973
7	0.2	80	30	0.7243
8	0.2	80	60	0.8760
9	0.2	80	90	0.8990
10	0.4	40	30	0.7352
11	0.4	40	60	0.9382
12	0.4	40	90	0.9879
13	0.4	60	30	0.9071
14	0.4	60	60	1.0511
15	0.4	60	90	1.1008
16	0.4	80	30	0.9390
17	0.4	80	60	1.2004
18	0.4	80	90	1.3205
19	0.6	40	30	0.9962
20	0.6	40	60	1.0872
21	0.6	40	90	1.1829
22	0.6	60	30	1.0936
23	0.6	60	60	1.2673
24	0.6	60	90	1.3171
25	0.6	80	30	1.1450
26	0.6	80	60	1.3628
27	0.6	80	90	1.4988

## Data Availability

The data presented in this study are available on request from the corresponding author.
